# Supplementary material for: Dietary folate intake and all-cause mortality and cardiovascular mortality in American adults with non-alcoholic fatty liver disease: Data from NHANES 2003 to 2018
Source: PLoS One. 2024 Nov 21;19(11):e0314148. doi: 10.1371/journal.pone.0314148 (PMC11581259; doi:10.1371/journal.pone.0314148)
Supplement: S1 File — (DOCX) [file pone.0314148.s001.docx]

Supporting information

| \| **S1 Table. Hazard Ratios for All-Cause Mortality Among Participants with NAFLD in NHANES 2003 to 2018** \| \| \| \| --- \| --- \| --- \| \|  \| **Dietary folate intake（μg/d）** \| \|  \| [Coef. (95%)] \| \| **Model 1** \| 0.999 (0.998-0.999) \| \| **Model 2** \| 0.999 (0.998-0.999) \| \| **Model 3** \| 0.999 (0.998-0.999) \|   **S2 Table. Hazard Ratios for CVD Mortality Among Participants with NAFLD in NHANES 2003 to 2018** | |
| --- | --- | --- | --- | --- | --- | --- | --- | --- | --- | --- | --- | --- | --- | --- |
|  | **Dietary folate intake（μg/d）** |
|  | [Coef. (95%)] |
| **Model 1** | 0.998 (0.997-0.999) |
| **Model 2** | 0.998 (0.996-0.999) |
| **Model 3** | 0.998 (0.997-1.000) |

| **S3 Table. Hazard Ratios for All-Cause Mortality Among Participants with NAFLD in NHANES 2003 to 2018(excluding participants who died within a two-year follow-up period)** | | | | | |
| --- | --- | --- | --- | --- | --- |
|  | **Dietary folate intake（μg/d）** | | | |  |
|  | **Quartile1**  **≤250μg/d** | **Quartile2**  **250.5-342.5μg/d** | **Quartile3**  **343-467μg/d** | **Quartile4**  **≥467.5μg/d** | Trend p-value |
| **Model 1** | 1.00 | 0.83 (0.62-1.10) | 0.76 (0.58-1.01) | 0.61 (0.46-0.82) | <0.001 |
| **Model 2** | 1.00 | 0.74 (0.56-0.98) | 0.62 (0.47-0.83) | 0.57 (0.42-0.77) | <0.001 |
| **Model 3** | 1.00 | 0.82 (0.60-1.11) | 0.80 (0.59-1.09) | 0.73 (0.52-1.02) | 0.084 |

| **S4 Table. Hazard Ratios for CVD Mortality Among Participants with NAFLD in NHANES 2003 to 2018(excluding participants who died within a two-year follow-up period)** | | | | | |
| --- | --- | --- | --- | --- | --- |
|  | **Dietary folate intake（μg/d）** | | | |  |
|  | **Quartile1**  **≤250μg/d** | **Quartile2**  **250.5-342.5μg/d** | **Quartile3**  **343-467μg/d** | **Quartile4**  **≥467.5μg/d** | Trend p-value |
| **Model 1** | 1.00 | 0.81 (0.50-1.31) | 0.71 (0.44-1.17) | 0.40 (0.24-0.69) | <0.001 |
| **Model 2** | 1.00 | 0.76 (0.45-1.26) | 0.64 (0.37-1.09) | 0.43 (0.23-0.77) | 0.005 |
| **Model 3** | 1.00 | 0.83 (0.46-1.51) | 0.88 (0.47-1.63) | 0.57 (0.28-1.14) | 0.162 |

| **S5 Table. Hazard Ratios for All-Cause Mortality Among Participants with NAFLD in NHANES 2003 to 2018 (excluding people who only had a single recollection of dietary folate intake)** | | | | | |
| --- | --- | --- | --- | --- | --- |
|  | **Dietary folate intake（μg/d）** | | | |  |
|  | **Quartile1**  **≤250μg/d** | **Quartile2**  **250.5-342.5μg/d** | **Quartile3**  **343-467μg/d** | **Quartile4**  **≥467.5μg/d** | Trend p-value |
| **Model 1** | 1.00 | 0.78 (0.59-1.03) | 0.71 (0.54-0.94) | 0.57 (0.43-0.75) | <0.001 |
| **Model 2** | 1.00 | 0.72 (0.54-0.94) | 0.60 (0.46-0.80) | 0.54 (0.40-0.73) | <0.001 |
| **Model 3** | 1.00 | 0.81 (0.61-1.07) | 0.77 (0.58-1.03) | 0.71 (0.52-0.97) | 0.036 |

| **S6 Table. Hazard Ratios for CVD Mortality Among Participants with NAFLD in NHANES 2003 to 2018 (excluding people who only had a single recollection of dietary folate intake)** | | | | | |
| --- | --- | --- | --- | --- | --- |
|  | **Dietary folate intake（μg/d）** | | | |  |
|  | **Quartile1**  **≤250μg/d** | **Quartile2**  **250.5-342.5μg/d** | **Quartile3**  **343-467μg/d** | **Quartile4**  **≥467.5μg/d** | Trend p-value |
| **Model 1** | 1.00 | 0.76 (0.47-1.22) | 0.65 (0.41-1.05) | 0.41 (0.25-0.68) | <0.001 |
| **Model 2** | 1.00 | 0.73 (0.45-1.19) | 0.60 (0.36-1.00) | 0.44 (0.25-0.76) | 0.003 |
| **Model 3** | 1.00 | 0.80 (0.46-1.40) | 0.83 (0.47-1.49) | 0.60 (0.31-1.15) | 0.166 |

| **S7 Table. Hazard Ratios for All-Cause Mortality Among Participants with NAFLD in NHANES 2003 to 2018 (removing NAFLD patients who died within two years of follow-up and had just one dietary recall)** | | | | | |
| --- | --- | --- | --- | --- | --- |
|  | **Dietary folate intake（μg/d）** | | | |  |
|  | **Quartile1**  **≤250μg/d** | **Quartile2**  **250.5-342.5μg/d** | **Quartile3**  **343-467μg/d** | **Quartile4**  **≥467.5μg/d** | Trend p-value |
| **Model 1** | 1.00 | 0.82 (0.61-1.11) | 0.74 (0.55-0.99) | 0.61 (0.45-0.82) | <0.001 |
| **Model 2** | 1.00 | 0.76 (0.57-1.02) | 0.63 (0.46-0.84) | 0.58 (0.42-0.80) | <0.001 |
| **Model 3** | 1.00 | 0.85 (0.62-1.16) | 0.80 (0.59-1.10) | 0.75 (0.53-1.06) | 0.108 |

| **S8 Table. Hazard Ratios for CVD Mortality Among Participants with NAFLD in NHANES 2003 to 2018 (removing NAFLD patients who died within two years of follow-up and had just one dietary recall)** | | | | | |
| --- | --- | --- | --- | --- | --- |
|  | **Dietary folate intake（μg/d）** | | | |  |
|  | **Quartile1**  **≤250μg/d** | **Quartile2**  **250.5-342.5μg/d** | **Quartile3**  **343-467μg/d** | **Quartile4**  **≥467.5μg/d** | Trend p-value |
| **Model 1** | 1.00 | 0.86 (0.52-1.43) | 0.72 (0.43-1.20) | 0.43 (0.25-0.74) | 0.001 |
| **Model 2** | 1.00 | 0.83 (0.49-1.41) | 0.67 (0.39-1.16) | 0.47 (0.25-0.86) | 0.010 |
| **Model 3** | 1.00 | 0.91 (0.49-1.70) | 0.94 (0.50-1.78) | 0.64 (0.31-1.30) | 0.264 |
